# On-Surface Molecular Recognition Driven by Chalcogen Bonding

**DOI:** 10.1021/jacsau.4c00325

**Published:** 2024-06-05

**Authors:** Luca Camilli, Conor Hogan, Deborah Romito, Luca Persichetti, Antonio Caporale, Maurizia Palummo, Marco Di Giovannantonio, Davide Bonifazi

**Affiliations:** † Department of Physics, University of Rome “Tor Vergata”, via della Ricerca Scientifica 1, 00133 Roma, Italy; ‡ CNR-Istituto di Struttura della Materia (CNR-ISM), 00133 Roma, Italy; § Department of Organic Chemistry, Faculty of Chemistry, University of Vienna, Währinger Straße 38, 1090 Vienna, Austria; ⊥ INFN, Department of Physics, University of Rome “Tor Vergata”, via della Ricerca Scientifica 1, 00133 Roma, Italy

**Keywords:** chalcogenazoles, chalcogen bonds, surface self-assembly, supramolecular chemistry, scanning tunneling microscopy, density functional theory, *ab initio* calculations

## Abstract

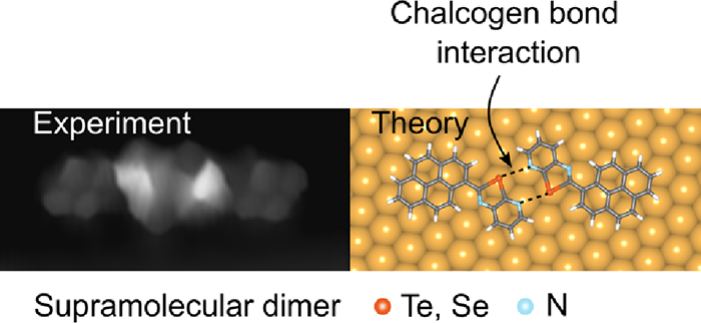

Chalcogen bonding interactions (ChBIs) have been widely employed to create ordered noncovalent assemblies in solids and liquids. Yet, their ability to engineer molecular self-assembly on surfaces has not been demonstrated. Here, we report the first demonstration of on-surface molecular recognition solely governed by ChBIs. Scanning tunneling microscopy and *ab initio* calculations reveal that a pyrenyl derivative can undergo noncovalent chiral dimerization on the Au(111) surface through double Ch···N interactions involving Te- or Se-containing chalcogenazolo pyridine motifs. In contrast, reference chalcogenazole counterparts lacking the pyridyl moiety fail to form regular self-assemblies on Au, resulting in disordered assemblies.

## Introduction

**Figure 1:**
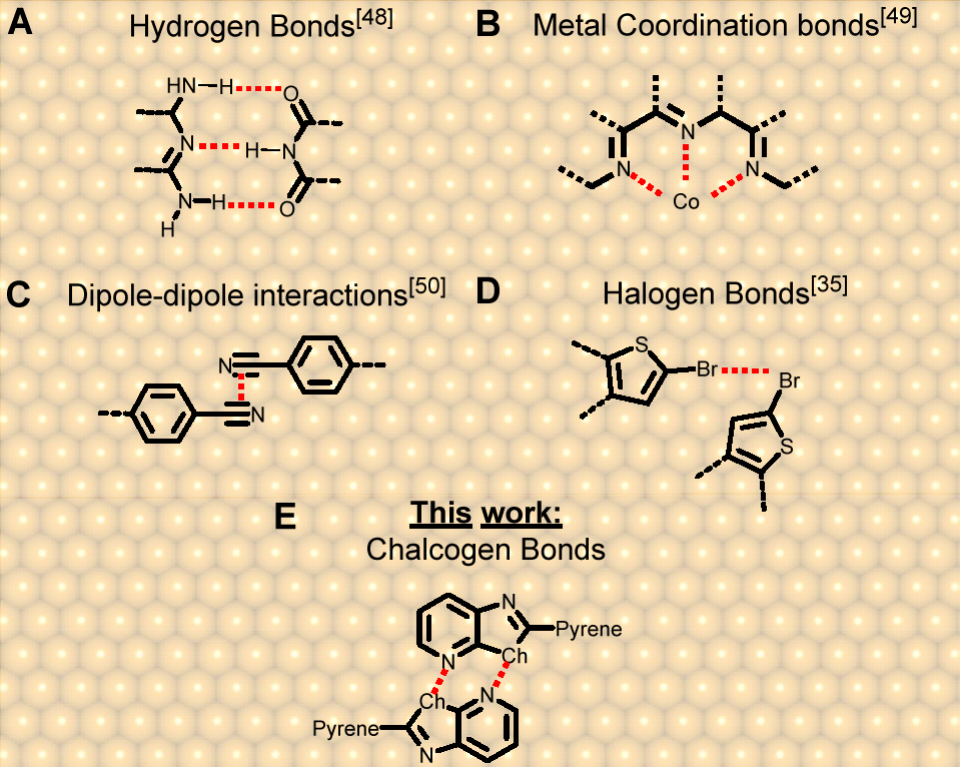
Schematic representations of the first examples of noncovalent molecular self-assembly at surfaces, respectively driven by (A) hydrogen bonds,^48^ (B) metal coordination bonds,^49^ (C) dipole–dipole interactions,^50^ (D) halogen bonds,^35^ and (E) ChBIs investigated in this work.

The manipulation of organic nanostructures on surfaces through the supramolecular approach has garnered substantial attention in recent decades.^1−7^ Among the various supramolecular interactions, H-bonding interactions have been extensively harnessed to foster the formation of highly organized two-dimensional (2D) networks.^8−12^ This has been followed by examples reporting coordination bonding^13−17^ and dipole–dipole^18,19^ interactions (Figure 1a–c). More recently, there has been a notable surge of interest in employing secondary bonding interactions (SBIs),^20^ which have a dual nature. Notably, from an electrostatic point of view,^21,22^ highly polarizable atoms are involved in effective SBIs through regions of depletion of electrons called σ-holes.^23^ The second aspect that drives flourishing interest for SBIs relies on the orbital mixing,^24^ described as n^2^(Y) → σ*(E–X) donation involving nonbonding electrons of the electron-rich Y atom, and the antibonding σ_E–X_^*^ on the E atom (with X being its covalent substituent). Within the category of SBIs, halogen bonding interactions^25−29^ have demonstrated their efficacy in creating regular supramolecular networks on surfaces,^30,31^ as revealed by scanning tunneling microscopy (STM) studies highlighting intermolecular Br···O,^32,33^ Br···Br,^34,35^ and Br···S^36^ halogen bonds (Figure 1d) governing the self-assembly. However, chalcogen bonding interactions (ChBIs)^37^ have not yet demonstrated comparable effectiveness on surfaces as they have in crystal engineering^38−42^ for developing functional materials,^43^ such as supramolecular semiconductors.^44,45^ Intermolecular Ch···N ChBs acting as the driving force for self-assembling chalcogenazole derivatives on surfaces, have only been theoretically explored in two recent studies.^46,47^ To the best of our knowledge, the role of ChB interactions in driving self-assembly on surfaces remained largely underinvestigated experimentally when compared to hydrogen bonds,^48^ metal coordination bonds,^49^ and dipole–dipole interactions,^50^ with only two reports suggesting the presence of ChB interactions and other noncovalent contacts.^18,51^

In this Letter, we combined bond-resolved STM (BRSTM) measurements with quantum chemistry calculations to elucidate the first example of ChB-driven molecular self-assembly on Au(111) using tailored recognition motifs that undergo self-assembly solely through ChB interactions. If one excludes the use of cationic heterocycles, one can note that outside a crystalline environment, the formation of such dimers is unprecedented. Indeed, even in solution, conclusive data demonstrating such self-assembly of neutral heterocycles have yet to be reported thus far.^52,53^ Building on earlier studies at the solid state, in which we have shown that chalcogenazolo pyridine (CGP) moieties persistently undergo self-assembly into dimers through double Ch···N interactions,^54−59^ we conjectured that the Se- and Te-bearing CGP motifs could also be exploited to govern molecular assemblies on surfaces (Figure 1e).^58^ With this aim, we designed and prepared pyrene-based CGP modules that could undergo dimerization through ChB-driven molecular recognition. Reference benzochalcogenazole congeners have also been investigated in which a C–H moiety has substituted the N-pyridyl atom and is, thus, not expected to establish any ChBIs (Section S1).

## Results

**Figure 2:**
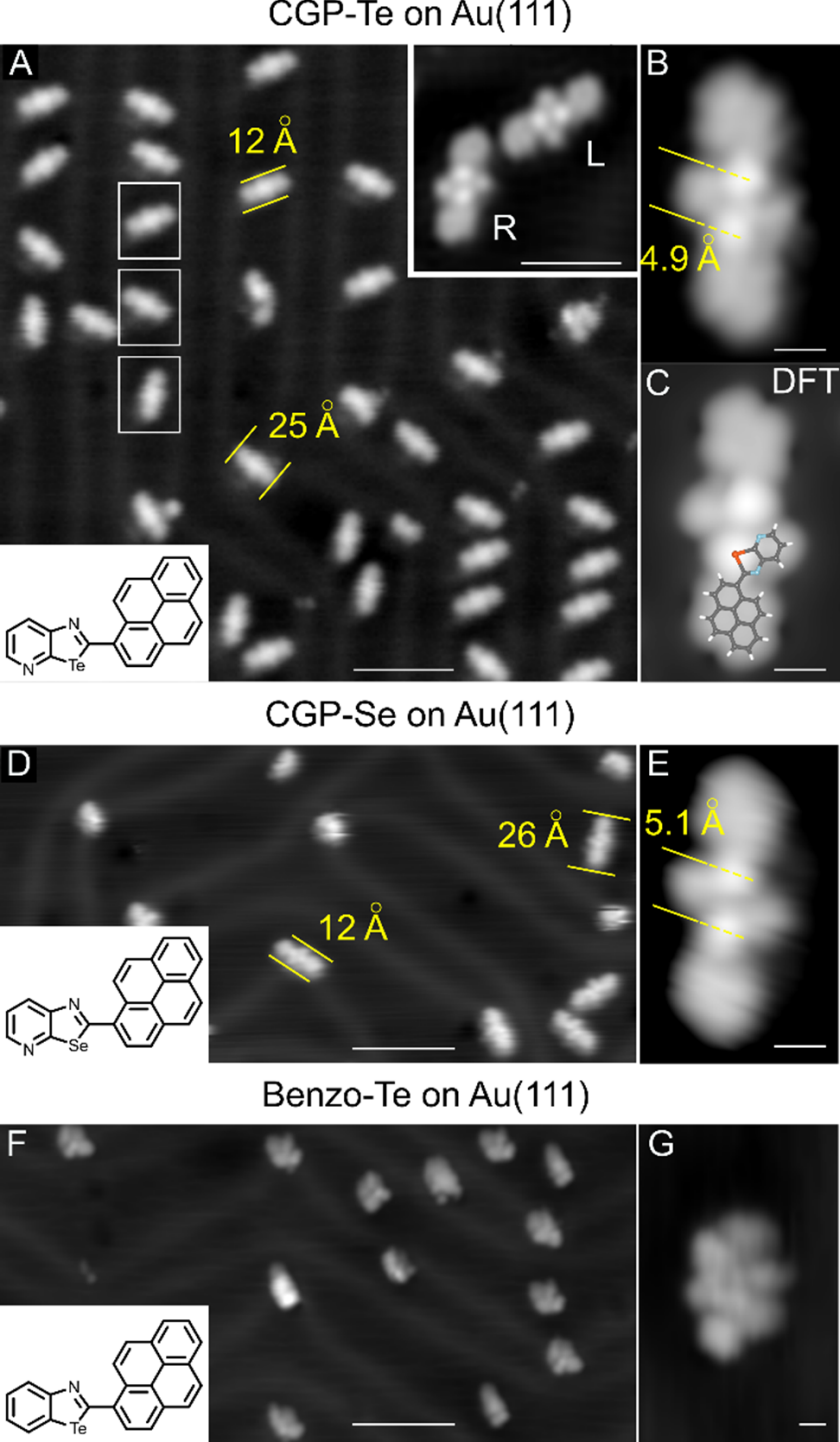
(A) STM image of **(CGP-Te)_2_** dimers on Au(111). The herringbone reconstruction of the Au substrate is also visible. In the rectangles, the 3-fold equivalent dimer orientations are highlighted. Tunneling current (*I*_t_) = 300 pA; tunneling bias (*V*_t_) = 1.000 V; *T* = 11 K. The top-right-corner inset shows two enantiomers; *I*_t_ = 250 pA; *V*_t_ = 0.500 V. (B) Experimental (*I*_t_ = 300 pA; *V*_t_= 0.630 V; *T* = 11 K) and (C) simulated (*V*_t_ = 0.630 V) STM image of an individual dimer. In the simulated image, the chemical structure of the monomer is also overlaid to display the strong Te-centered signal. (D) STM image of self-assembled **(CGP-Se)_2_** dimers on Au(111). *I*_t_ = 300 pA; *V*_t_ = 0.800 V; *T* = 8.5 K. (E) STM image of an individual dimer. *I*_t_ = 150 pA; *V*_t_ = 0.100 V; *T* = 8.5 K. (F) STM image of the reference tellurazole molecules deposited on Au(111). Kinetic aggregates of various shapes and sizes can be observed. *I*_t_ = 400 pA; *V*_t_ = 1.000 V; *T* = 11 K. (G) Detail of a molecular assembly. *I*_t_ = 400 pA; *V*_t_ = 1.000 V; *T* = 11 K. Scale bars: 5 nm in (A), (D), and (F); 0.5 nm in (B), (C), (E), and (G); and 2 nm in the inset of (A).

A constant-current STM image of a Au(111) crystal after room temperature deposition of **CGP-Te** in vacuum is shown in Figure 2a. Isolated, straight structures with a length of 2.5 ± 0.1 nm, displaying 6-fold rotational symmetry as exemplified by the three white rectangles, are usually found on the face-centered cubic (fcc) regions of the reconstructed Au(111) surface. Given the inherent asymmetry of the molecules on surfaces, two enantiomers for each dimer were found (R and L, inset in Figure 2a) with a relative distribution of around 50% (see Section S4). A close-up view of one of these structures highlights the presence of two bright-contrast spots in the middle region 4.9 ± 0.2 Å from each other (Figure 2b). The simulated STM image (Figure 2c), obtained by density functional theory (DFT) calculations of the adsorbed dimer in its most stable geometry (see below), is in perfect agreement with the experimental images. Thus, it is reasonable to conclude that the observed structure is a dimer **(CGP-Te)**_**2**_, in which the two middle bright-contrast spots correspond to the Te atoms belonging to the **CGP-Te** moieties. Notably, the two monomers are oriented head-to-head with the Te atoms facing the *N*-pyridyl atom of the neighboring molecule, supporting the presence of the ChB-driven association. As observed in the STM images of **CGP-Se** (Figures 2d,e and S5), dimeric **(CGP-Se)**_**2**_ structures are also formed. Dimers are also observed when **CGP-Te** molecules are deposited on a Ag(110) held at room temperature, which generalizes our findings (Figure S4). On the other hand, when reference **benzo-Te** molecules are deposited on Au, only irregular aggregate-type species are observed (Figure 2f,g). This confirms our hypothesis that the absence of the *N*-pyridyl atoms prevents dimer formation since the double ChBIs can no longer be established. Similarly, reference **benzo-Se** modules do not undergo dimerization; only individual molecules are observed (Figure S2).

**Figure 3:**
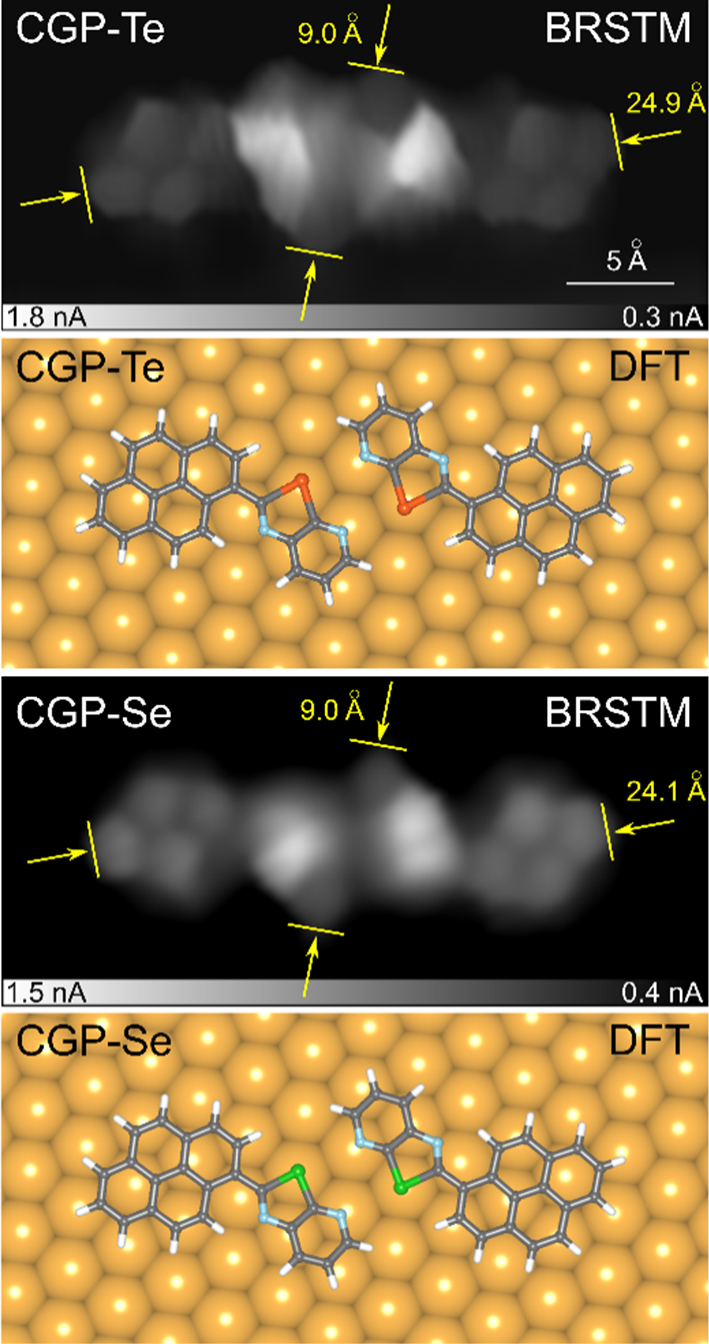
From top to bottom: BRSTM image (constant height, *V*_t_ = 5 mV, *T* = 8.7 K) and DFT relaxed geometrical model of the individual **(CGP-Te)**_**2**_ and **(CGP-Se)**_**2**_ dimers.

To unequivocally disclose the chemical structure of the self-assembled **(CGP-Te)**_**2**_ and **(CGP-Se)**_**2**_ dimers, constant-height STM experiments with a CO-functionalized tip were performed. Figure 3 reports so-called bond-resolved (BR)STM images of the individual dimers and the respective relaxed geometrical model computed by DFT. The pyrene and pyridyl moieties are distinguishable in the experimental images, while a robust electronic signal arises around the Ch atoms. The total length of the dimers can be measured as 2.49 and 2.41 nm for **(CGP-Te)**_**2**_ and **(CGP-Se)**_**2**_, respectively, while their width is 9.0 Å in both cases. DFT geometry optimizations reveal that the two Ch atoms in the dimer lie at on-top positions with respect to the underlying Au lattice and bind to second-nearest-neighbor (second NN) atoms (Figure 3). Indeed, the optimized Te···Te (Se···Se) distance of 4.94 Å (4.82 Å) in the gas-phase dimer matches well with the Au second nn distance of 5.05 Å (Table S1). The calculated Te···Te (Se···Se) distance of 5.01 Å (4.94 Å) in the adsorbed dimer is in notable agreement with the distance measured between the two bright-contrast spots observed in the STM image (Figure 2b,e).

**Figure 4:**
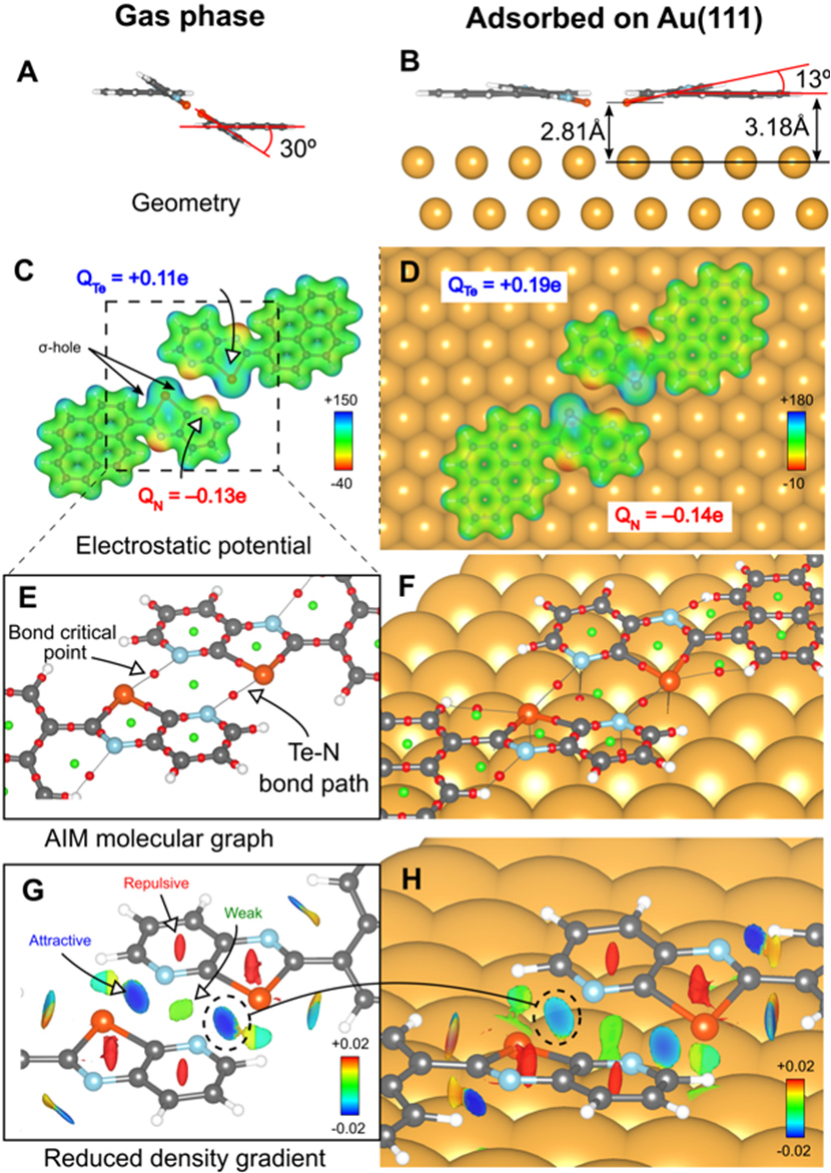
DFT analysis of ChBIs in a (**CGP-Te)**_**2**_ dimer. (A, B) Gas phase and adsorption geometries (side view). The tilt angle of the chalcogenazole moiety with respect to the pyrene unit is shown. (C, D) Electrostatic potential (in au) superimposed on a charge density isosurface (ρ = 0.025 au). Atomic charges *Q* are reported for Te and N. (E, F) Molecular graph showing bond paths (dotted lines), bond critical points in red, and ring critical points in green. For clarity, bond paths between the dimer and substrate are not shown, except for Te···Au and N···Au. (G, H) Reduced density gradient (on the 0.5 au isosurface) showing the noncovalent ChBIs; blue and red regions indicate attractive and repulsive interactions, respectively; the dashed circles highlight the attractive interaction at the bond critical point between Te and N atoms.

The formation of dimers (or lack thereof) is determined by the interaction energy, Δ*E*_int_, between the monomers. In the gas phase, Δ*E*_int_ is defined as the energy gain in forming the dimer from its constituent fixed-geometry fragments (see Section S10). The computed values (Table S1) indicate exothermic processes of −9.2 and −6.2 kcal mol^–1^ for **CGP-Te** and **CGP-Se**, respectively. The corresponding geometry for **(CGP-Te)**_**2**_ is plotted in Figure 4a, showing alignment of the chalcogenazole rings Te···N distances of 3.0 Å (Table S1). For comparison, the Δ*E*_int_ value for chalcogenadiazole dimers is about −17 and −7 kcal mol^–1^ for Te- and Se-containing congeners (Ch···N distances are 2.6 and 2.9 Å), respectively.^59,60^ When adsorbed on Au(111), surface strain and relaxation effects must also be considered when computing the Δ*E*_int_. A derivation of the on-surface interaction energy is given in Sections S10 and S17. We calculated Δ*E*_int_ values at −5.7 and −3.9 kcal mol^–1^ for **(CGP-Te)**_**2**_ and (**CGP-Se)**_**2**_, respectively, suggesting that the dimer formation remains favorable, albeit weaker, also on Au(111). It is worth pointing out that the interaction between **CGP-Te** or **CGP-Se** monomers in the dimer is, in fact, still strong enough to allow us to manipulate a dimer with the STM tip without breaking it apart (Figure S3). In contrast, the Δ*E*_int_ value for dimers of reference **benzo-Te** is revealed to be +1.1 kcal mol^–1^ on the surface, consistent with the experimental observation that no dimers are formed. A deeper analysis of Δ*E*_int_ is given in Section S17.

DFT was then used to analyze the chemical nature of the ChBIs. Previous theoretical studies elucidating the character of ChBIs in homodimers^24,47,59−62^ included molecular electrostatic potential maps, reduced density gradient (RDG) plots,^63^ and quantum theory of atoms-in-molecules (AIM)^64^ and natural bond order calculations. Moreover, energy decomposition analyses^59,61^ reveal that electrostatic effects can contribute significantly (up to 58%) to Δ*E*_int_ in chalcogenadiazole dimers, while the orbital mixing component can be as large as 41% in telluradiazoles.^62^ A favorable Δ*E*_int_ combined with the electrostatic potential map, RDG, and AIM analyses is sufficient to confirm the presence and the noncovalent nature of ChBIs in these systems.^37,47^

Figure 4c shows the molecular electrostatic potential for **CGP-Te** superimposed on a charge density isosurface. The blue maxima appearing at the extensions of the Te back-bonds indicate σ-holes. Their alignment with the red minima, corresponding to the N-lone-pairs, is a typical feature in ChBIs.^65^ After Au adsorption (Figure 4d), the σ-hole character remains present, although it appears tilted toward the surface (see also Figures 4b and S7) due to the strong Te···Au (and N···Au) interaction. The subsequent misalignment of the σ-hole and N-lone-pair binding the two monomers is consistent with the reduction of the calculated Δ*E*_int_ after adsorption. The AIM analysis (Figure 4e) reveals bond paths along both Te···N contacts containing bond critical points, constituting evidence of a ChBI. Notably, both features persist in the Au-adsorbed **(CGP-Te)**_**2**_ (Figure 4f) and (**CGP-Se)**_**2**_ (Figure S8) dimers. Finally, RDG plots (Figure 4g) explicitly identify the Te···N interaction as noncovalent.^63^ The blue color of the RDG isosurface, centered at the Te···N bond, indicates that the Te···N interaction is attractive, as expected from the sign of Δ*E*_int_. The Te···N interaction persists in the adsorbed system (Figure 4h), although the less intense color indicates a weaker contact. The interaction appears weaker again in **(CGP-Se)**_**2**_ dimers (Figure S8), consistent with an expected smaller orbital contribution.^62^ These conclusions are further supported by calculations of the charge density difference (Figure S9) and molecular projected density of states (Figure S13). In contrast, similar analyses for **Benzo-Te** reveal a fundamentally different, weaker interaction (Figure S10 and Table S2).

The total energy and charge density analyses support our hypothesis that the ChBIs govern the surface-confined dimerization of the pyrenyl derivatives and confirm the STM results. This occurs despite a considerable dimer···Au interaction which determines the adsorption site, flattened molecular geometry, and azimuthal orientation (Figure S6). Although a large van der Waals component anchors the molecule to the surface, a Te···Au bond is identifiable (Figures S7 and S9). The total charge transfer from **(CGP-Te)**_**2**_ to the Au(111) surface is 0.36e, mainly coming from the Te atom (Δ*Q*_Te_ = +0.08e, see Figure 4b). Such an increase might naively imply a *larger* electrostatic interaction after adsorption. However, the computed *reduction* in interaction energy suggests that the charge depletion is associated with the Te lone pair aligned toward the Au surface. It thus has little influence on the in-plane intermolecular ChBI.

## Conclusions

In conclusion, our study comprehensively explores nanostructure self-assembly on surfaces guided by ChBI molecular recognition. Specifically, we utilized chalcogenazolo pyridine (with Ch = Se and Te) moieties to create supramolecular chiral dimers through double ChBIs on Au(111). The combination of BRSTM measurements and quantum chemistry calculations clarified the formation of these dimers, characterized by distinct 6-fold rotational symmetry and upheld by nonbonding interactions between Ch atoms and adjacent pyridine moieties. On-surface ChBI was also demonstrated on other substrates (Figure S4) and for other moieties (Figure S10).^46,47^ In contrast, reference chalcogenazole compounds lacking the *N*-pyridyl atom and thus incapable of establishing ChBIs do not form dimers but assemble into irregularly shaped kinetic aggregates. Charge density analysis of the **(CGP-Te)**_**2**_ dimer confirmed the attractive noncovalent nature of Te···N interactions, which persist when assembled on Au(111). The distinctive feature of ChBIs, characterized by their strong orbital contribution, leads us to anticipate that our findings will pave the way for designing and fabricating precise supramolecular nanostructures on surfaces with tailored semiconducting properties.^41^ Ultimately, this study not only expands our comprehension of supramolecular interactions but also sheds light on a promising avenue for future research in the bottom-up engineering of two-dimensional (2D) monolayered supramolecular chalcogenide-type materials as we delve into the novel role of ChBIs in surface-based molecular recognition.

## Methods

### Synthesis

The syntheses of **CGP-Te**, **CGP-Se**, and **Benzo-Te** were performed following previous literature reports.^54,56^ For details on the protocols and structural characterization, please see the Supporting Information.

### Surface Studies

All molecules studied here were sublimated in ultrahigh vacuum from a commercial evaporator (Kentax) onto an Au(111) single crystal that was previously cleaned following standard Ar^+^ sputter/anneal cleaning cycles. During sublimation, the Au(111) substrate was held at room temperature, with the pressure in the chamber being below 2 × 10^–10^ mbar. All STM measurements were performed using a commercial Infinity system from Scienta Omicron held at temperatures between 11 and 8 K (the exact temperature is specified in the text for each reported STM image). The STM images were calibrated so that the measured Au lattice constant would coincide with the one from the simulation after geometry optimization (lattice constant: 4.122 Å). A CO-functionalized W tip was used for BRSTM. BRSTM images were collected in constant height mode with a low tunneling bias (5 mV). All experimental images were analyzed using the Gwyddion software.^66^

Surface studies Calculations were performed using DFT in a planewave/pseudopotential framework implemented in the quantum-ESPRESSO (QE) code.^67^ The Perdew–Burke–Ernzerhof (PBE) exchange–correlation functional was used,^68^ and van der Waals interactions were accounted for semiempirically via the Grimme-D3 method with Becke-Johnson damping.^69^ Ultrasoft Rappe-Rabe-Joannopoulos-Kaxiras (RRJK) pseudopotentials were used (cutoff 45/360 Ry). Gas phase geometries were computed in a 45 × 35 × 25 Å^3^ cell. The optimal monomer geometry was determined by rotating the pyrene group about the bond to the azole unit until an energy minimum was reached and then free relaxation was performed. The substrate was modeled using a four-layer Au(111) slab (*a*_0_ = 4.12 Å), whose backmost two layers were fied. Monomer and dimer adsorption were modeled within supercells of size 46.6 Å × 20.2 Å, ensuring an intermolecular separation of at least 12 Å and a vacuum spacing between periodically repeating images of 20 Å. Gamma-point sampling with a Marzari-Vanderbilt smearing of 0.1 eV was used throughout.^70^ Geometry optimizations were performed using a tight 5 meV Å^–1^ threshold. Several initial geometries for monomer adsorption were tested. The most stable geometry features the chalcogen atom at the on-top site. By testing the azimuthal energy dependence, we identified the optimal orientation of having the pyrene groups aligned along the Au atom rows. This configuration was then used to construct possible geometries for the dimer, including chalcogen atoms at the nearest and second nearest neighbor sites and for different lateral offsets and azimuthal orientations. The most stable geometries are the ones reported in the main text. The RDG^71^ and electrostatic maps were also computed using QE. Analysis of atomic charges and quantum theory of AIM paths were performed with the critic2 code^72^ using all-electron charge densities computed with the QE package. RDG and molecular electrostatic potential (MEP) maps were visualized using VESTA.^9^ The MEP maps are plotted on a relatively high value of charge density isosurface (ρ = 0.025 au; Figure S5 for a comparison with the standard plot at ρ = 0.001 au) to reveal the σ-holes also in the dimer and the adsorbed systems. RDG isosurfaces are colorized using the product of the charge density and sign of the second eigenvalue of the electron density Hessian matrix in the range [−0.02:0.02] au.^73^ STM images were computed using the Tersoff-Hamann approximation.^74^ Atomic charges were computed using the Voronoi deformation density (VDD) method.^75^

## Supporting Information Available

Experimental details and additional data (PDF)
