# Supplementary material for: On-Surface Molecular Recognition Driven by Chalcogen Bonding
Source: JACS Au. 2024 Jun 5;4(6):2115–21. doi: 10.1021/jacsau.4c00325 (PMC11200221; doi:10.1021/jacsau.4c00325)
Supplement: Supplementary file 1 — au4c00325_si_001.pdf [file au4c00325_si_001.pdf]

Supporting Information material for
On-surface Molecular Recognition driven by Chalcogen Bonding

Luca Camilli[a] §,*, Conor Hogan[b,a] §,*, Deborah Romito[c] §, Luca Persichetti[a], Antonio Caporale[a], Maurizia Palummo[d], Marco Di Giovannantonio[b] and Davide Bonifazi[c]*

[a] Department of Physics, University of Rome "Tor Vergata", via della Ricerca Scientifica 1, 00133 Roma (Italy)

[b] CNR-Istituto di Struttura della Materia, (CNR-ISM), 00133 Roma (Italy)

[c] Department of Organic Chemistry, Faculty of Chemistry, University of Vienna, Währinger Straße 38, 1090 Vienna (Austria)

[d] INFN, Department of Physics, University of Rome "Tor Vergata", via della Ricerca Scientifica 1, 00133 Roma (Italy)

Contents

1. Synthesis 2

Synthesis of 2-(pyren-1-yl)benzo[δ][1,3]selenazole Benzo-Se 2

Synthesis of 2-(pyren-1-yl)benzo[δ][1,3]tellurazole Benzo-Te 2

Synthesis of 2-(pyren-1-yl)-[1,3]selenazolo[5,4-β]pyridine CGP-Se 4

Synthesis of 2-(pyren-1-yl)-[1,3]tellurazolo[5,4-β]pyridine CGP-Te 5

2. Surface studies 6
3. Computational methodology 6
4. Chirality of CGP-Te dimers on Au(111) 6
5. STM of reference molecule Benzo-Se 8
6. Additional bond-resolved scanning tunnelling microscopy (BRSTM) images 8
7. CGP-Te dimer formation on Ag(110) 9
8. Monolayer CGP-Se on Au(111) 10
9. Thermal stability of CGP-Te dimers 10
10. Molecular adsorption, binding and interaction energies 11
11. Optimized geometry of CGP-Te on Au 14
12. Electrostatic potential maps of CGP-Te 15
13. DFT analysis of chalcogen bonding in the CGP-Se dimer 16
14. Electron density redistribution following CGP-Te dimer formation 17
15. Bonding analysis in Benzo-Te 18
16. Chalcogenadiazole (diazole-Te) dimer formation on Au(111) 20
17. Interaction energy analysis 21
18. Molecular projected density of states (PDOS) 24

Methodology

1. Synthesis

The molecules studied in this work have been prepared according to a synthetic protocol previously published[1] (Scheme S1).

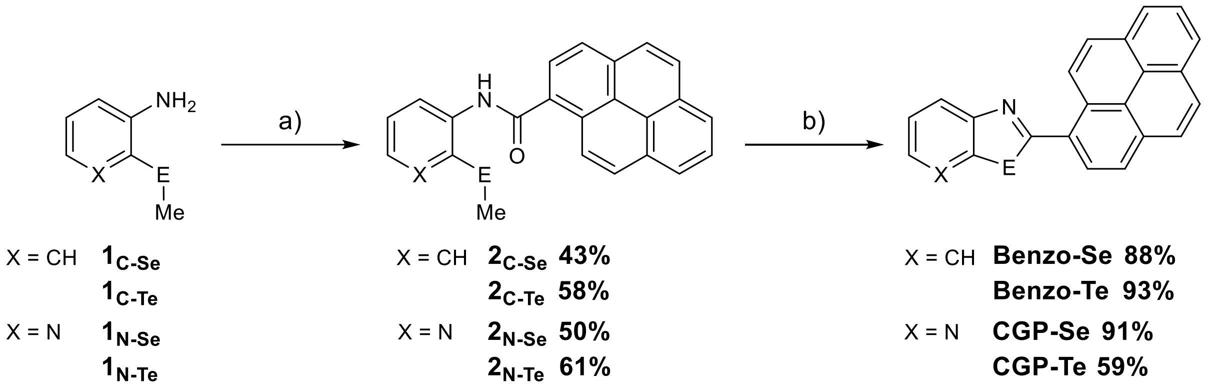

Synthesis of 2-(pyren-1-yl)benzo[δ][1,3]selenazole Benzo-Se

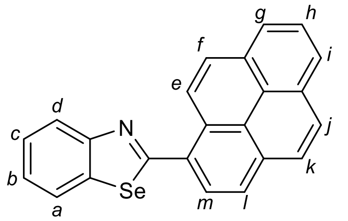

Benzo-Se

To a solution of *N*-(2-(methylselanyl)phenyl)pyrene-1-carboxamide **2**$_{C\text{-}Se}$ (228 mg, 0.55 mmol) and NEt_3 (366 mg, 0.46 mL, 3.3 mmol) in dry dioxane (11 mL) under anhydrous condition, $POCl_3$ (184 mg, 0.10 mL, 1.1 mmol) was added dropwise. The reaction was heated to reflux and stirred for 5 h. The resulting mixture was diluted with $CHCl_3$ (100 mL), washed with a saturated aqueous $NaHCO_3$ solution (30 mL), then with water (30 mL), brine (30 mL), and dried over $MgSO_4$. The solvents were removed under reduced pressure. The crude was purified by silica gel chromatography ($CHCl_3$) to give pure **Benzo-Se** as a bright yellow solid (184 mg, 88%).

mp = 130-132 °C; FTIR (ATR): ν (cm^{-1}): 3215, 1643, 1584, 1522, 1501, 1431, 1387, 1304, 1213, 1186, 1067, 941, 817, 746, 708; ^{1}H NMR (400 MHz, CD_2Cl_2) δ: 9.41 (d, J = 9.6 Hz, 1H, H_f), 8.40 (d, J = 8.0 Hz, 1H), 8.30-8.23 (m, 5H), 8.20 (d, J = 9.0 Hz, 1H), 8.14 – 8.05 (m, 3H), 7.59 (ddd, J = 8.5, 7.3, 1.3 Hz, 1H), 7.41 (ddd, J = 8.5, 7.3, 1.3 Hz, 1H); ^{13}C NMR (100 MHz, CD_2Cl_2) δ: 172.5, 156.3, 139.7, 132.7, 131.4, 130.9, 129.9, 129.4, 129.2, 129.0, 128.2, 127.3, 126.5, 126.4, 126.1, 125.8, 125.4, 125.2, 125.1, 124.84, 124.8, 124.4; HRMS (ESI): *m/z* cacld for $C_{23}H_{13}N^{80}Se{+}H^+$: 380.0318 [$M$+H]$^+$; found: 380.0312.

Synthesis of 2-(pyren-1-yl)benzo[δ][1,3]tellurazole Benzo-Te

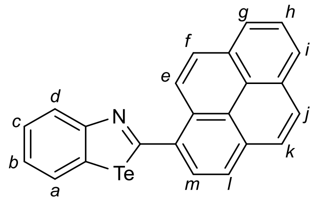

Benzo-Te

To a solution of *N*-(2-(methyltellanyl)phenyl)pyrene-1-carboxamide $\mathbf{2}_{\mathbf{C\text{-}Te}}$ (349 mg, 0.75 mmol) and NEt_3 (455 mg, 0.63 mL, 4.5 mmol) in dry dioxane (15 mL) under anhydrous condition, $POCl_3$ (225 mg, 0.14 mL, 1.5 mmol) was added dropwise. The reaction was heated to reflux and stirred for 2 h. The resulting mixture was diluted with $CHCl_3$ (150 mL), washed with a saturated aqueous $NaHCO_3$ solution (30 mL), water (30 mL), brine (30 mL), and dried over $MgSO_4$. The solvents were removed under reduced pressure. The crude was purified by silica gel chromatography ($CHCl_3$) to give pure **Benzo-Te** as a bright yellow solid (300 mg, 93%).

mp = 134-136 °C; FTIR (ATR): *v* (cm^{-1}): 3021, 1580, 1503, 1429, 1290, 1173, 1063, 1018, 924, 839, 756; ^1H NMR (400 MHz, CD_2Cl_2) δ: 9.37 (d, J = 9.4 Hz, 1H, H_f), 8.38 (ddd, J = 8.1, 1.2, 0.5 Hz, 1H), 8.29-8.15 (m, 6H), 8.10-8.03 (m, 3H), 7.59 (ddd, J = 8.1, 7.2, 1.3 Hz, 1H), 7.28 (ddd, J = 7.8, 7.2, 1.2 Hz, 1H); ^{13}C NMR (100 MHz, CD_2Cl_2) δ: 173.4, 162.5, 136.6, 134.9, 132.4, 131.6, 131.5, 131.0, 130.3, 129.1, 128.8, 127.3, 127.1, 126.9, 126.8, 126.5, 126.0, 125.6, 125.2, 125.1, 125.07, 125.0, 124.4; HRMS (ESI): *m/z* cacld for $C_{23}H_{13}N^{127}Te+H^+$: 426.0157 [*M*+H]+; found: 426.0154.

Synthesis of 2-(pyren-1-yl)-[1,3]selenazolo[5,4-β]pyridine CGP-Se

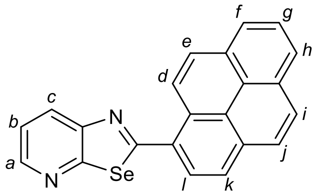

CGP-Se

To a solution of *N*-(2-(methylselanyl)pyridin-3-yl)pyrene-1-carboxamide $\mathbf{2}_{\mathbf{N\text{-}Se}}$ (117 mg, 0.28 mmol) and NEt_3 (366 mg, 0.47 mL, 3.6 mmol) in dry dioxane (6 mL) under anhydrous condition, $POCl_3$ (184 mg, 0.11 mL, 1.2 mmol) was added dropwise. The reaction was heated to reflux and stirred overnight. The resulting mixture was diluted with $CHCl_3$ (50 mL), washed with a saturated aqueous $NaHCO_3$ solution (20 mL), water (20 mL), brine (20 mL), and dried over Na_2SO_4. The solvents were removed under reduced pressure. The crude was purified by silica gel chromatography ($CHCl_3$/MeOH 99:1) to give pure **CGP-Se** as a bright yellow solid (98 mg, 91%).

mp = 156-158 °C; FTIR (ATR): v (cm^{-1}): 3038, 1576, 1512, 1373, 1207, 1186, 945, 820, 839, 795, 735, 630; ^{1}H NMR (300 MHz, CD_2Cl_2) δ: 9.38 (d, J = 9.5 Hz, 1H, H_e), 8.57 (dd, J = 4.7, 1.6 Hz, 1H, H_a), 8.46 (dd, J = 8.2, 1.6 Hz, 1H, H_c), 8.40 (d, J = 8.0 Hz, 1H, H_i), 8.31 – 8.19 (m, 5H, $H_{d,f,h,k,l}$), 8.15 – 8.06 (m, 2H, $H_{g,j}$), 7.53 (dd, J = 8.2, 4.7 Hz, 1H, H_b); ^{13}C NMR (75 MHz, CD_2Cl_2) δ: 174.4, 164.4, 150.2, 146.7, 133.0, 131.5, 131.4, 130.8, 129.8, 129.6, 129.4, 129.3, 128.3, 127.3, 126.6, 126.3, 125.9, 125.1, 125.0, 124.9, 124.3, 121.6; HRMS (ESI): *m/z* cacld for $C_{22}H_{12}N_2{}^{78}Se+H^+$: 385.0239 $[M+H]^+$; found: 384.0282.

Synthesis of 2-(pyren-1-yl)-[1,3]tellurazolo[5,4-β]pyridine CGP-Te

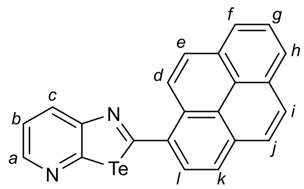

CGP-Te

To a solution of *N*-(2-(methyltellanyl)pyridin-3-yl)pyrene-1-carboxamide $\mathbf{2_{N\text{-}Te}}$ (93 mg, 0.2 mmol) and NEt_3 (243 mg, 0.34 mL, 2.4 mmol) in dry dioxane (4 mL) under anhydrous condition, $POCl_3$ (123 mg, 0.08 mL, 0.8 mmol) was added dropwise. The reaction was heated to reflux and stirred overnight. The resulting mixture was diluted with $CHCl_3$ (50 mL), washed with a saturated aqueous $NaHCO_3$ solution (20 mL), water (20 mL), brine (20 mL), and dried over Na_2SO_4. The solvents were removed under reduced pressure. The crude was purified by silica gel chromatography ($CHCl_3$/MeOH 99:1) to give pure **CGP-Te** as a bright yellow solid (51 mg, 59%).

mp = 188-190 °C. FTIR (ATR): ν (cm^{-1}): 1573, 1535, 1473, 1365, 1207, 1180, 934, 837, 791, 578, 478; ^{1}H NMR (500 MHz, CD_2Cl_2) δ: 9.35 (d, J = 9.4 Hz, 1H, H_e), 8.54 (dd, J = 4.6, 1.6 Hz, 1H, H_a), 8.49 (dd, J = 8.0, 1.6 Hz, 1H, H_c), 8.31 – 8.06 (m, 5H, $H_{d,f,g,h,j}$), 8.21 (d, J = 8.9 Hz, 1H, H_k), 8.14 – 8.06 (m, 2H, $H_{i,l}$), 7.53 (dd, J = 8.0, 4.6 Hz, 1H, H_b); ^{13}C NMR (125 MHz, CD_2Cl_2) δ: 177.7, 165.4, 158.3, 146.4, 135.0, 132.7, 132.5, 131.5, 131.0, 130.4, 129.3, 129.1, 127.4, 127.1, 126.6, 126.2, 125.8, 125.2, 125.05, 125.04, 124.5, 121.9; HRMS (ESI): *m/z* cacld for $C_{22}H_{12}N_2{}^{125}Te+H^+$: 435.0135 $[M+H]^+$; found: 435.0134.

2. Surface studies

The details of the surface investigation were described in the manuscript.

3. Computational methodology

As for the experimental investigation on surfaces, the calculation methods were extensively described in the manuscript.

Supplementary Experimental Results

4. Chirality of CGP-Te dimers on Au(111)

By looking carefully at our STM images of CGP-Te dimers on Au(111) one can notice the existence of two enantiomers (Fig. S1). Statistically, after analyzing several images taken in different locations on a given sample for several samples, we found a ratio of 1.01 between the two species, as expected since they are degenerate.

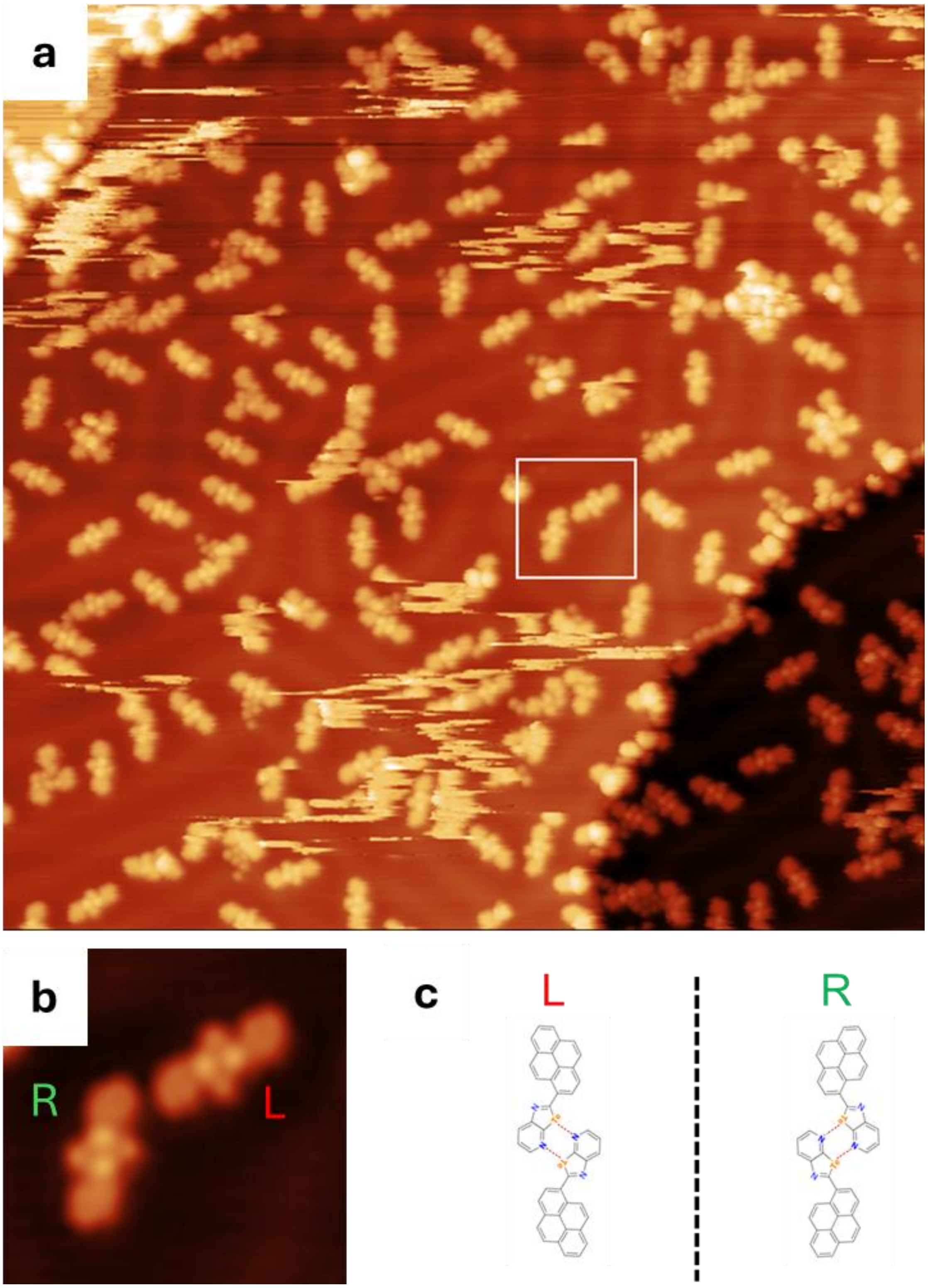

Figure S1. a) 40x40 nm^2 Constant-current scanning tunnelling microscopy image collected at 11.1 K of CGP-Te deposited on Au(111). Two enantiomers can be found, as exemplified by the blow-up shown in panel b (zoom of the white square in parent: 250 pA; panel a). Tunnelling bias voltage: 0.5 V. c) Sketch of the two enantiomers.

5. STM of reference molecule Benzo-Se

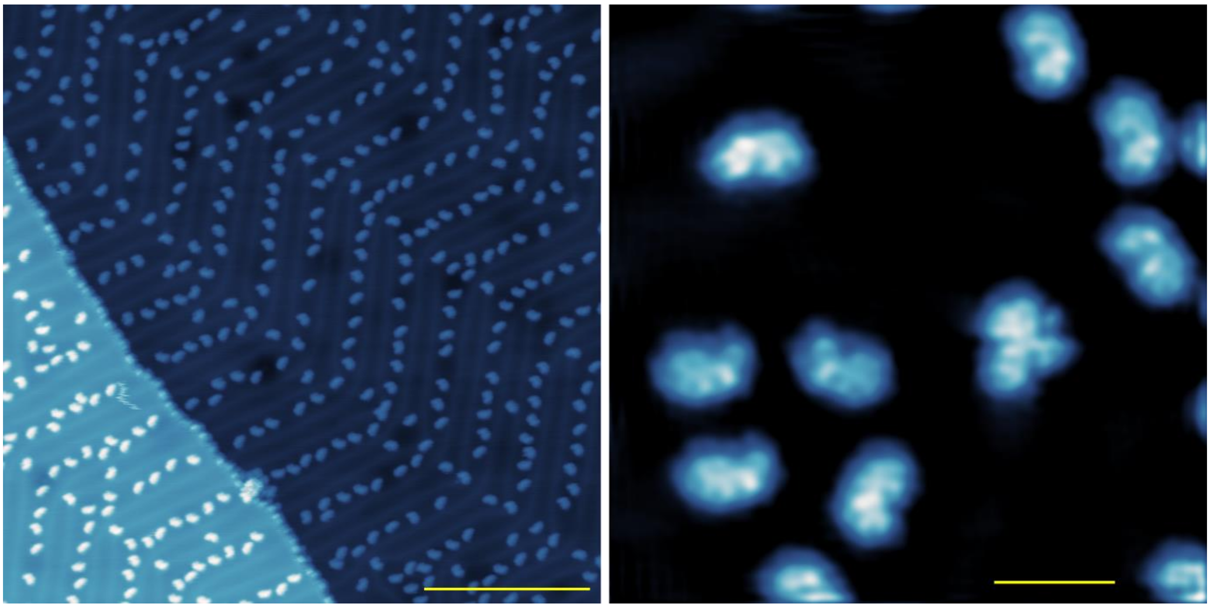

Figure S2. Constant-current scanning tunnelling microscopy (STM) images of reference **Benzo-Se** molecules on Au(111) at T=9.5 K. The monomer molecules self-assemble along the herringbone reconstruction of the substrate but do not form dimers and remain well isolated. Scale bars: 20 nm (left panel) and 2 nm (right panel). Tunnelling current: 0.1 nA (left panel) and 0.08 nA (right panel); tunnelling bias: 1.000 V (left panel) and 0.100 V (right panel).

6. Additional bond-resolved scanning tunnelling microscopy (BRSTM) images

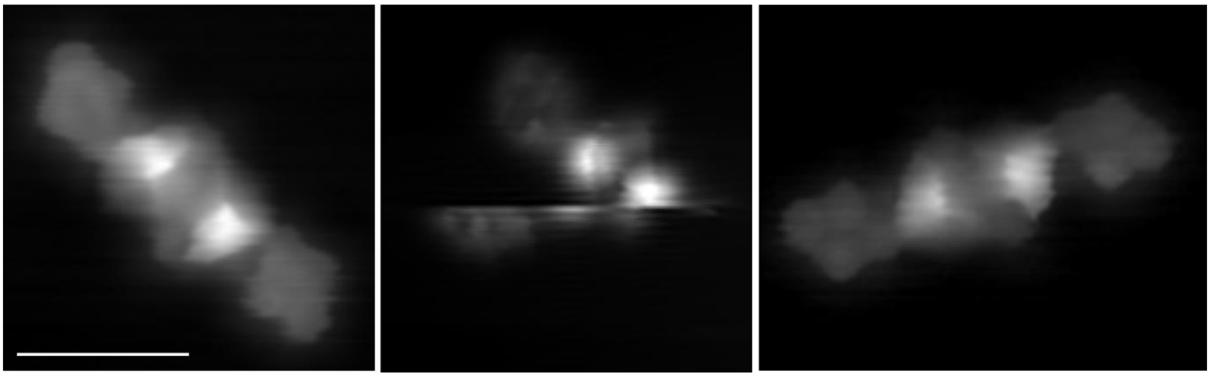

Figure S3. BRSTM images collected on the same **CGP-Te** dimer at T= 8.7 K. The dimer has rotated during the scan (see middle panel) due to tip-dimer interaction but remains intact after rotation, showing the stability of the dimer. The rotation angle of 60 degrees shows both the relatively strong bonding with the substrate, which forces Te atoms to be on top position with respect to the Au atoms underneath.

The image to the left was acquired in constant height mode with a CO-functionalized tip with 0.005 V, while the ones at the centre and to the right with 0.000 V. Scale bar: 1 nm.

7. CGP-Te dimer formation on Ag(110)

To generalize our findings, we have attempted to use chalcogen bonding interaction to form dimers on a metal surface other than Au. As shown in Fig. S3, after deposition of **CGP-Te** molecules on a clean Ag(110) substrate held at room temperature, a number of **CGP-Te** dimers are observed from STM characterization.

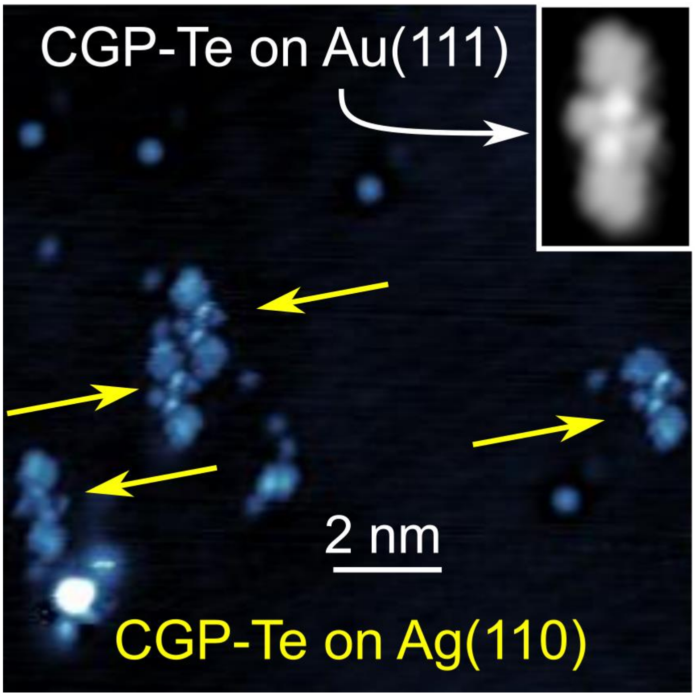

Figure S4. Scanning tunnelling microscopy image of **CGP-Te** deposited on Ag(110). **CGP-Te** molecules were sublimated in ultra-high vacuum on the Ag(110) substrate held at room temperature. Four dimers can be spotted in this region. The image was taken at 12 K with an etched W tip. Tunnelling current: 150 pA; tunnelling bias: 1.0 V. For comparison, the inset in the top right corner shows the **CGP-Te** dimer on Au(111) already displayed in panel B of Figure 2 in the manuscript.

8. Monolayer CGP-Se on Au(111)

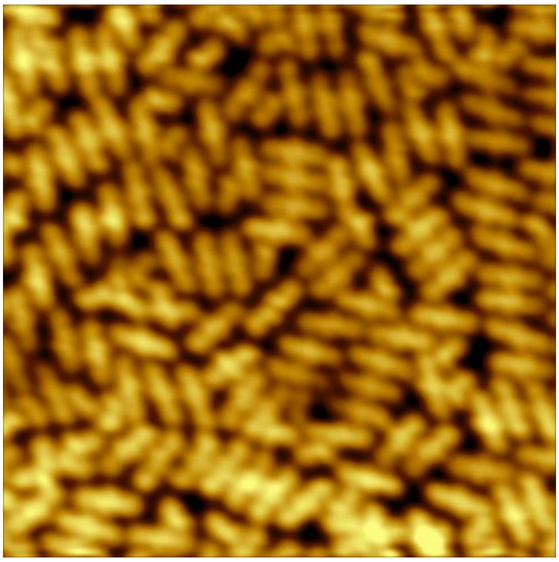

Figure S5. 20x20 nm^2 scanning tunnelling microscopy image of **CGP-Se** deposited on Au(111). **CGP-Se** molecules were sublimated in ultra-high vacuum on the Au(111) substrate held at room temperature. The coverage is close to 1 ML. The image was taken at 9.5 K with an etched W tip. Tunnelling current: 100 pA; tunnelling bias: 1.033 V.

9. Thermal stability of CGP-Te dimers

To test the thermal stability of the CGP-Te dimers, after its deposition, we annealed a sample of CGP-Te/Au(111) to 50 °C prior to performing STM analysis. The results of the analysis are displayed in Fig. S6. No noticeable differences can be found with respect to a sample that was not annealed.

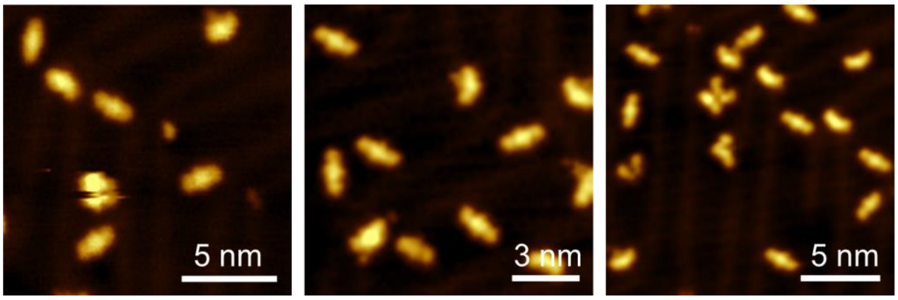

Figure S6. Constant-current STM images of a sample of CGP-Te/Au(111) that was annealed to 50 C after deposition at room temperature. Tunnelling current: 30 pA; bias voltage: 1.0 V; acquisition temperature: 10.9 K.

Supplementary Theoretical Results

10. Molecular adsorption, binding and interaction energies

Adsorption, binding and interaction energies are defined in the following way. We adopt a naming convention whereby $E^{tot}_{A/B}$ means the total energy of the interface formed by molecule A adsorbed on substrate B, while $E^{tot}_{C,D}$ means the total energy of system C when it is in the geometry (phase) D.

The *gas-phase dimer binding energy* is the energy gained by forming the dimer from freestanding monomers:

$$\Delta E^{b}_{dimer,gas} := E^{tot}_{dimer,gas} - 2E^{tot}_{monomer,gas} \quad (1)$$

where the terms on the right-hand side are the DFT total energies of the isolated dimer and monomer in their fully relaxed, ground state geometry.

The *gas-phase dimer interaction energy* is the energy gained by forming the dimer from its constituent fragments (i.e., the monomers fixed in the geometries they take in the dimer):

$$\Delta E^{int}_{dimer,gas} := E^{tot}_{dimer,gas} - E^{tot}_{fragment\ 1,gas} - E^{tot}_{fragment\ 2,gas} \quad (2)$$

The difference between binding and interaction energies is the induced strain (deformation) energy.

We now introduce the corresponding expressions for the molecules adsorbed on the surface. If the molecule-substrate interaction is so weak that the substrate geometry does not change, the binding and interaction energies can be defined in a trivial way.[2] In general, however, the substrate deformation energy must be taken into account.

The *surface dimer interaction energy* is the energy gained by forming the dimer from its fragments:

$$\Delta E^{int}_{dimer,surf} := E^{tot}_{dimer/Au} - E^{tot}_{fragment\ 1/Au} - E^{tot}_{fragment\ 2/Au} + E^{tot}_{Au,dimer} \quad (3)$$

where $E^{tot}_{molecule/Au}$ is the total energy of a complete interface, slab plus molecule, system (molecule = dimer, monomer, fragment, etc), and $E^{tot}_{Au,dimer}$ is the energy of the substrate (slab) alone, frozen in the geometry reached when the dimer is adsorbed (adsorbed dimer phase). In the case of **CGP-Te** on Au(111), $E^{tot}_{Au,dimer}$ and $E^{tot}_{Au,clean}$ (the total energy of the clean Au surface) differ by 1.8 kcal/mol, which is indeed comparable to $\Delta E^{int}_{dimer,surf}$, i.e. it must be taken into account explicitly.

The *surface dimer binding energy* is the energy gained by bringing two adsorbed monomers together to form the adsorbed dimer. It is defined as:

$$\Delta E^{b}_{dimer,surf} := E^{tot}_{dimer/Au} - E^{tot}_{Au,dimer} - 2(E^{tot}_{monomer/Au} - E^{tot}_{Au,monomer}) \qquad (4)$$

where the last term is the energy of the frozen slab (substrate) alone, when the monomer is adsorbed on top (adsorbed monomer phase).

Finally, the *adsorption energy* is always defined in terms of the relaxed, gas-phase monomer:

$$\Delta E^{ads}_{monomer} := E^{tot}_{monomer/Au} - E^{tot}_{Au,clean} - E^{tot}_{monomer,gas} \qquad (5)$$

$$\Delta E^{ads}_{dimer} := E^{tot}_{dimer/Au} - E^{tot}_{Au,clean} - 2E^{tot}_{monomer,gas} \qquad (6)$$

where $E^{tot}_{Au,clean}$ is the total energy of the clean Au surface and $E^{tot}_{molecule/Au}$ that of the complete interface (slab+molecule). In the limit of negligible surface strain, the adsorption and binding energies are simply related as:

$$\Delta E^{b}_{dimer,surf} \approx \Delta E^{ads}_{dimer} - 2\Delta E^{ads}_{monomer} \qquad (7)$$

	**Gas phase**					**Adsorbed on surface**					
	ΔE^{b}	ΔE^{int}	Ch-N	Ch-Ch	N-N	ΔE^{b}	ΔE^{int}	ΔE^{ads}	Ch-N	Ch-Ch	N-N
**CGP-Te**	–8.94	-9.24	3.01	4.94	3.56	-4.21	-5.68	-138.9	3.17, 3.24	5.01	3.76
**CGP-Se**	-6.03	-6.20	2.99	4.82	3.37	-3.18	-3.85	-123.7	3.15, 3.28	4.94	3.58
**Benzo-Te**	-5.12	-5.21	-	6.31	-	+1.45	+1.11	-136.1	-	5.26	-
**Diazole-Te**	-18.01	-21.23	2.55	3.79	2.67	-9.94	-19.99	-64.8	2.50, 2.50	3.68	2.63

Table S1. Binding (ΔE^{b}), interaction (ΔE^{int}), and adsorption (ΔE^{ads}) energies (in kcal/mol) related to dimer formation in the gas phase and adsorbed on the Au(111) surface. Ch(Te, Se)-N and Ch-Ch distances in Å. The corresponding 1st and 2nd nearest neighbour Au-Au distances are 2.92 Å and 5.05 Å. CGP = chalcogenazolo pyridine; Benzo = benzochalcogenazole; diazole = chalcogenadiazole.

11.Optimized geometry of CGP-Te on Au

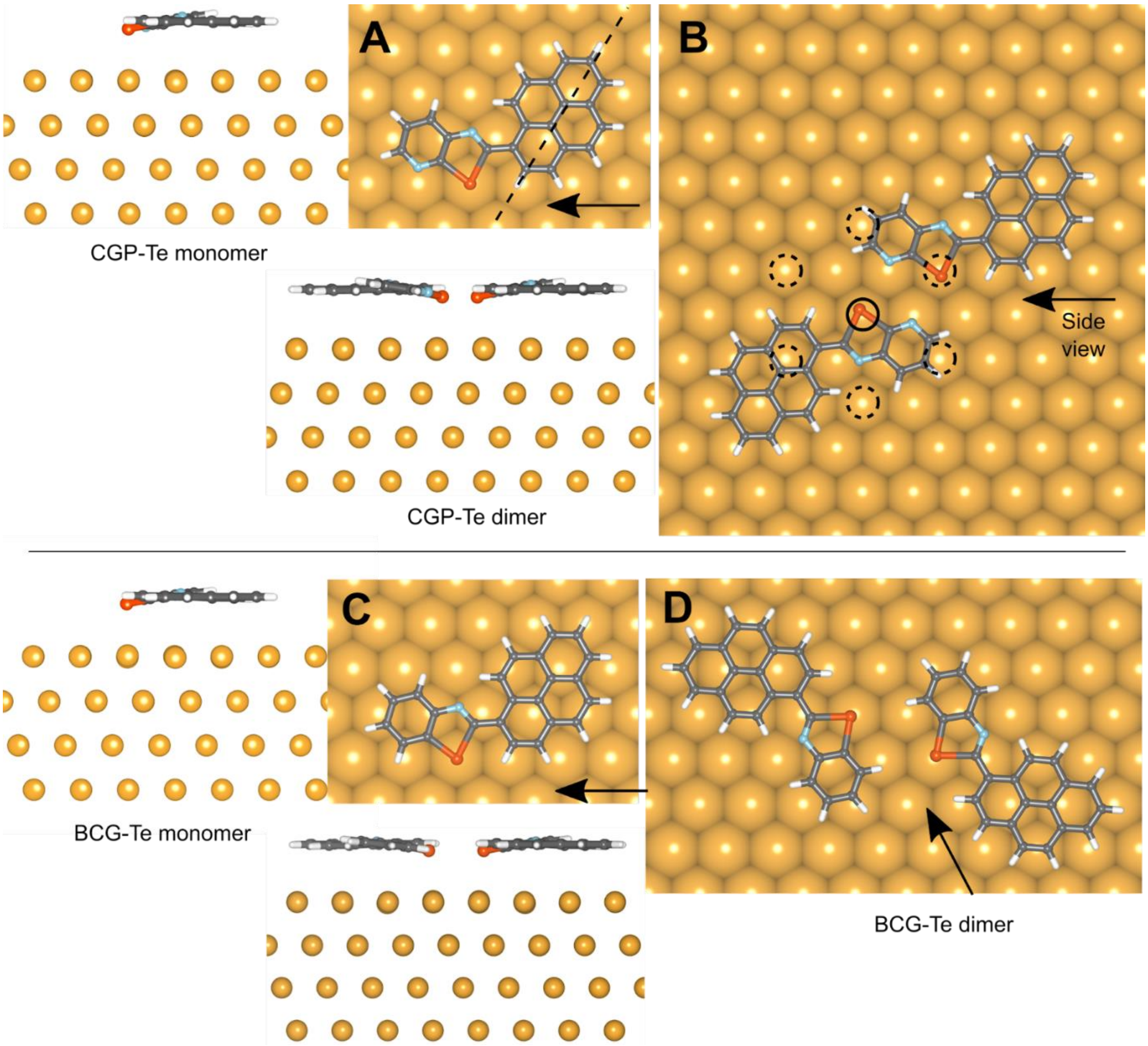

Figure S6. (A, B) Geometry of **CGP-Te** monomer and dimer adsorbed on Au(111). The arrows indicate the direction of the side views. In (B), the six-fold possible orientations of the dimer are indicated. The circles indicated Te on Au matching. The **CGP-Se** geometry is almost identical. (C, D) Geometry of reference **Benzo-Te** tellurazole ("BCG-Te") molecule adsorption.

12. Electrostatic potential maps of CGP-Te

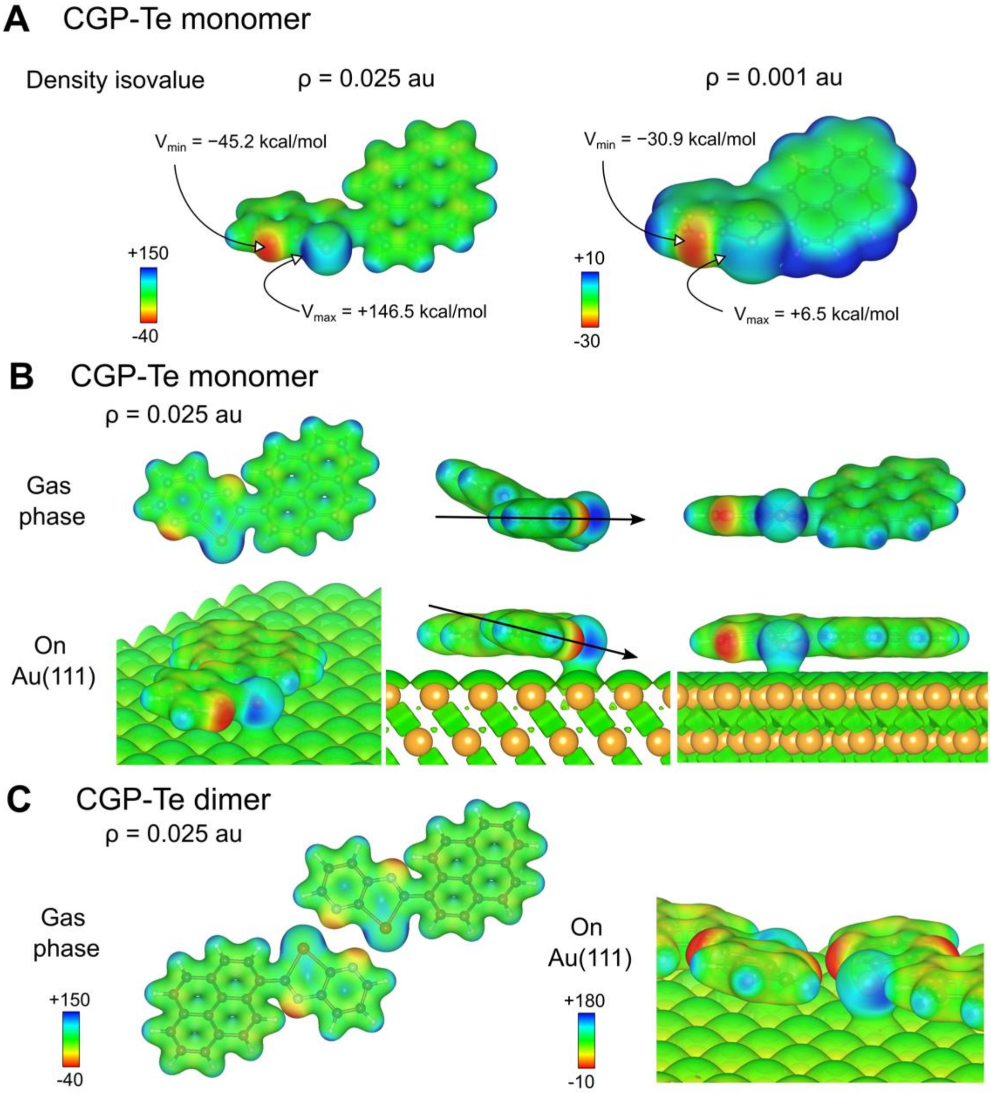

Figure S7. (A) Molecular electrostatic potential (MEP) maps of the **CGP-Te** monomer superimposed on the ρ = 0.025 au and ρ = 0.001 au charge density isosurface, respectively. (B, C) MEPs of the **CGP-Te** monomer and dimer, in gas phase and after adsorption on Au(111), plotted on the ρ = 0.025 au charge density isosurface. Different views are presented to highlight the interaction with the surface. The arrows roughly indicate the tilt of the sigma hole.

13. DFT analysis of chalcogen bonding in the CGP-Se dimer

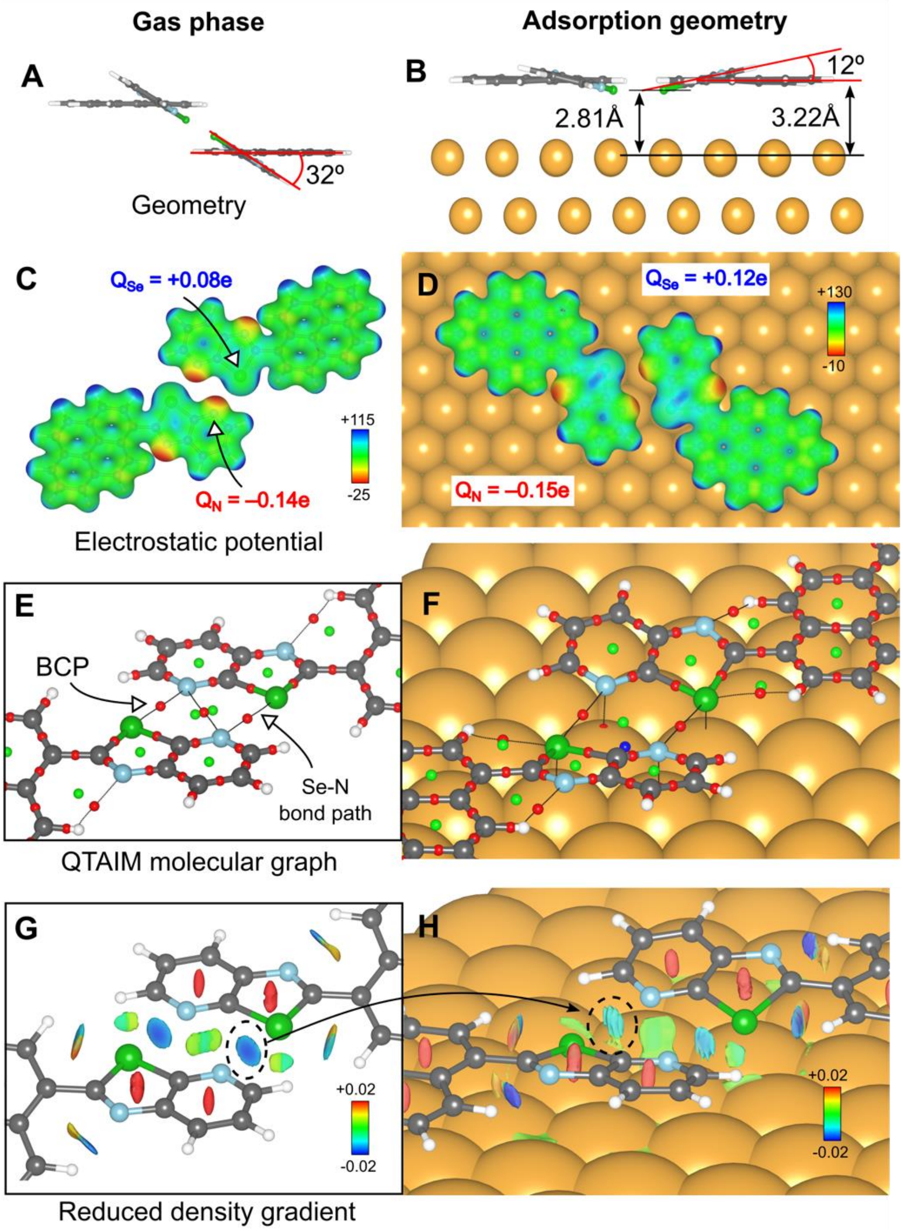

Figure S8. DFT analysis of chalcogen bonding in the **CGP-Se** dimer. (A, B) Gas phase and adsorption geometries (side view). The tilt angle of the azole with respect to the pyrene ring is indicated. (C, D) Electrostatic potential (in au) superimposed on a charge density (ρ = 0.025 au) isosurface. Atomic charges Q are reported for Se and N. Total charge transfer to the surface ΔQ = 0.22e. (E, F) Molecular graph showing bond paths (dotted lines), bond critical points (BCP) in red, and ring critical points (in green). For clarity, critical points and bond paths between the dimer and substrate are not shown, except for Se-Au and N-Au. An additional N-N bond path is found for the gas phase **CGP-Se** dimer that is not present in **CGP-Te**. (G, H) Reduced density gradient (on the 0.5 au isosurface); blue and red indicate attractive and repulsive interactions, respectively. The dashed circle highlights the attractive non-covalent interaction between Se and N, that persists in the adsorbed dimer but with a weaker intensity.

14. Electron density redistribution following CGP-Te dimer formation

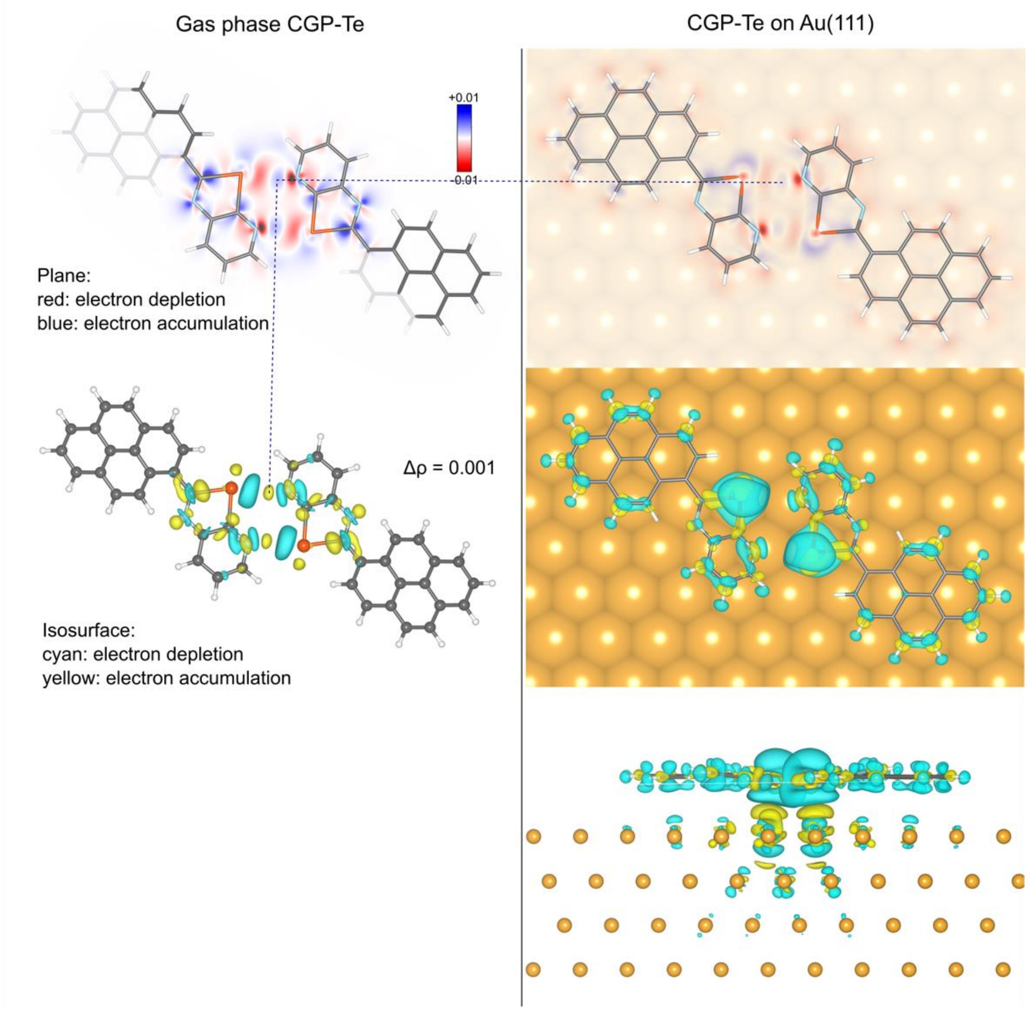

Figure S9. Electronic charge density difference plots of the **CGP-Te** dimer, computed using (for gas phase, left) Δρ = ρ(dimer) - ρ(fragment1) - ρ(fragment2) or (for adsorbed phase, right) Δρ = ρ(dimer/Au) - ρ(fragment1) - ρ(fragment2) - ρ(Au). Density ρ given in a.u. The top row shows Δρ as a contour map on a horizontal plane containing the Te atoms; middle and bottom rows show Δρ isosurfaces (top and side views). The dotted line indicates a weak area of electron accumulation between the Te and N atoms. Note that the intermolecular charge redistribution appears only along the Te-N axes, and persists following adsorption. Polarization is notable within the azole rings. A relatively large transfer of electrons from Te to Au is visible in the side profile, consistent with the VDD analysis reported in the main text and in Fig. 4.

15. Bonding analysis in Benzo-Te

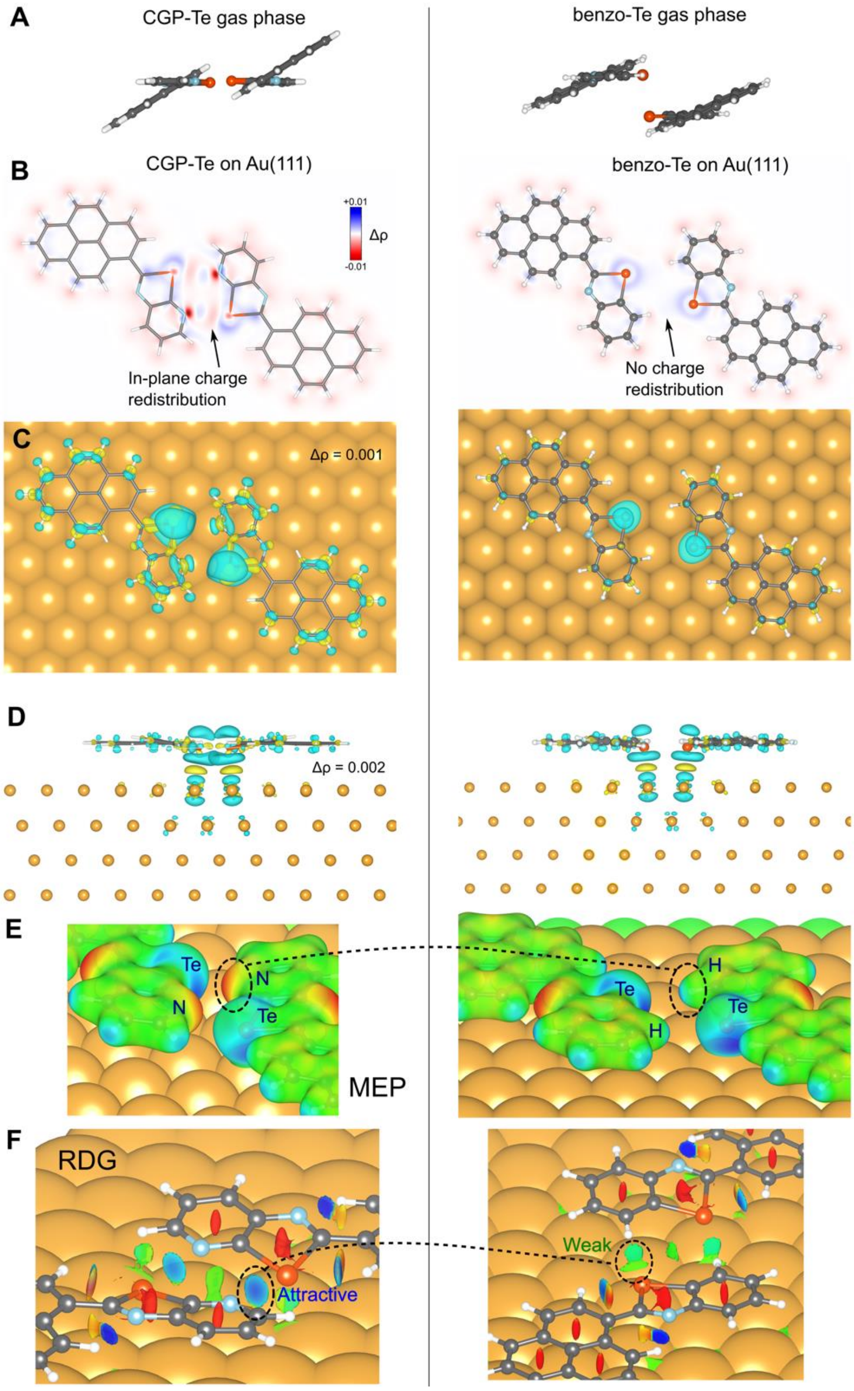

Figure S10. Chalogen bonding in the **CGP-Te** dimer (left), compared with the weak interaction within the **Benzo-Te** dimer (right). (A) Gas phase geometries. (B) Electronic charge density difference plots: $\Delta\rho = \rho(\text{dimer/Au}) - \rho(\text{fragment1}) - \rho(\text{fragment2}) - \rho(\text{Au})$, as a contour map on the horizontal plane containing the Te atoms. Density ρ is in a.u. (C,D) As (A) but for single isovalues of Δρ; top and side views. (E) Molecular electrostatic potential (MEP) maps (in au) superimposed on a charge density ($\rho = 0.025$ au) isosurface. (F) Reduced density gradient (RDG; on the 0.5 au isosurface); blue and red indicate attractive and repulsive interactions, respectively. The dotted lines in (E) and (F) highlight the

difference between the moderately attractive Te-N chalcogen bond and the weakly attractive Te-H interaction.

16. Chalcogenadiazole (diazole-Te) dimer formation on Au(111)

Chalcogen bonding between chalcogenadiazole molecules has been deeply studied in the literature[3-5] (e.g. Scheiner et al; Michalczyk et al; Wang et al**).** In Fig. S9 we demonstrate that, like for **CGP-Te**, chalcogen bonding in a simple chalcogenadiazole dimer (diazole-Te) persists following adsorption on Au(111). While monomer adsorption (A) occurs in the on-top (Te on Au) position with a vertical geometry, the intermolecular interaction within the dimer induces a flat geometry. In contrast to **CGP-Te**, lattice matching of (Te, Te) occurs with respect to the 1st nearest neighbour Au-Au distance. Adsorption, binding, and interaction energies are reported in Table S1. Due to the short Ch-N bonds, the interaction and binding energies are much higher than **CGP-Te**, although the adsorption energy is much lower. Charge density difference plots (C), molecular electrostatic potential maps (D), and reduced density gradient maps (E), all indicate the presence of directional, non-covalent Te-N bonds.

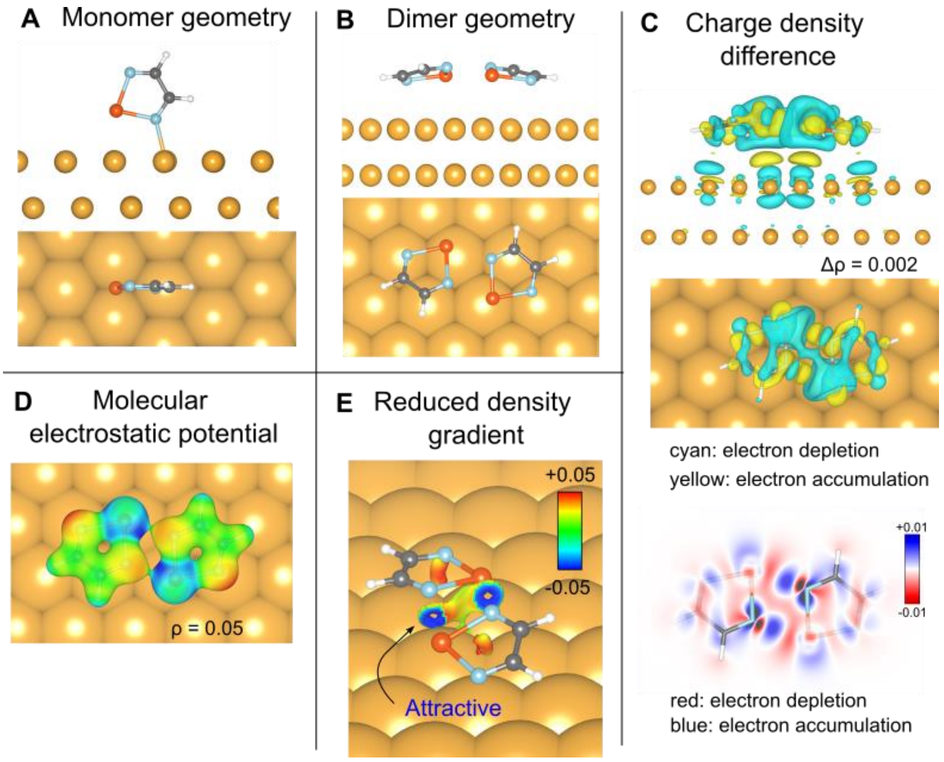

Figure S11. Chalcogen bonding in a chalcogenadiazole dimer adsorbed on Au(111). (A) Adsorption geometry of the monomer and (B) dimer. (C) Electronic charge density difference plots: $\Delta\rho = \rho(\text{dimer/Au}) - \rho(\text{fragment1}) - \rho(\text{fragment2}) - \rho(\text{Au})$, as a contour map on the horizontal plane containing the Te atoms and for a single isovalue of Δρ. Density ρ is in a.u. (D) Molecular electrostatic potential maps (in au) superimposed on a charge density (ρ = 0.05 au) isosurface. (E) Reduced density gradient (on the 0.5 au isosurface); blue and red indicate attractive and repulsive interactions, respectively.

17. Interaction energy analysis

The dimer interaction energy quantifies the attraction between each fragment in a dimer in gas phase (Eq. 2) or when adsorbed on the surface (Eq. 3). By computing $\Delta E^{int}_{dimer,peeled}$, i.e. the interaction energy of the dimer in the "peeled-off" geometry (in its adsorbed geometry with the substrate removed),

$$\Delta E^{int}_{dimer,peeled} := E^{tot}_{dimer,peeled} - E^{tot}_{fragment\ 1,peeled} - E^{tot}_{fragment\ 2,peeled} \quad (7)$$

the change in the interaction energy due to the presence of the substrate can be analysed further. Firstly, we define the *dimer deformation energy*:

$$\Delta E^{int,def}_{dimer,surf} := \Delta E^{int}_{dimer,peeled} - \Delta E^{int}_{dimer,gas} \quad (8)$$

which describes the penalty incurred in moving from the gas phase dimer geometry to the "peeled-off" dimer geometry. $\Delta E^{int,def}_{dimer,surf}$ quantifies the effect of the misalignment of the Te-N orbitals (azole/pyridine rings) due to twisting of the azole towards the surface, the modified Te-N distance due to lattice matching, and the relative rotation of azole and pyrrole rings after adsorption.

Secondly, we define

$$\Delta E^{int,CT}_{dimer,surf} := \Delta E^{int}_{dimer,surf} - \Delta E^{int}_{dimer,peeled} \quad (9)$$

where $\Delta E^{int,CT}_{dimer,surf}$ is the energy change (penalty) in the dimer interaction energy incurred due to its electronic interaction with the surface, e.g. from charge transfer (CT), orbital mixing with Au states, etc.

Note that the surface deformation energy is excluded from the dimer interaction energy; instead, it is implicit in the dimer *binding* energy.

Finally, we decompose the surface dimer interaction energy into a dispersion (vdW-D3) and a non-dispersion term:

$$\Delta E^{int}_{dimer,surf} = \Delta E^{int,non-disp}_{dimer,surf} + \Delta E^{int,disp}_{dimer,surf} \quad (10)$$

	$\Delta E^{int}_{dimer,gas}$	$\Delta E^{int,def}_{dimer,surf}$	$\Delta E^{int}_{dimer,peeled}$	$\Delta E^{int,CT}_{dimer,surf}$	$\Delta E^{int}_{dimer,surf}$	$\Delta E^{int,disp}_{dimer,surf}$	$\Delta E^{int,non-disp}_{dimer,surf}$
**CGP-Te**	–9.24	+1.81	–7.43	+1.75	–5.68	–3.83	–1.85
**CGP-Se**	–6.20	+1.26	–4.94	+1.09	–3.85	–3.15	–0.70
**Benzo-Te**	–5.21	+2.65	–2.56	+3.67	+1.11	–1.83	+2.94
Diazole-Te	–21.23	+0.62	–20.60	+0.62	–19.99	–3.13	–16.86

Table S2. Contributions to the dimer interaction energy, in kcal/mol. See Eqs. 3, 7—10 for definitions of each quantity.

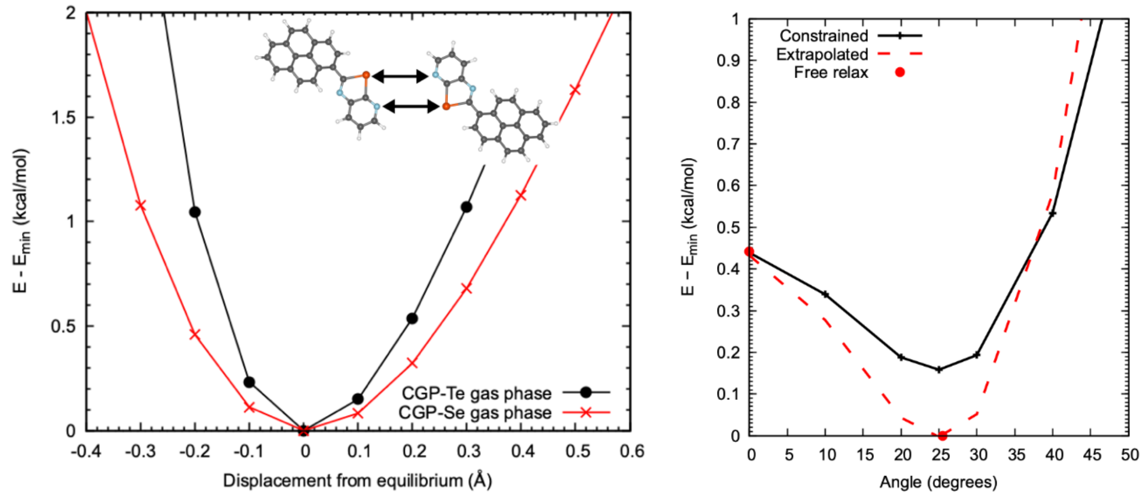

Figure S12. Left: change in dimer interaction energy with monomer separation. Energy is given with respect to that of the equilibrium geometry in the gas phase. Right: energy variation for the **CGP-Te** monomer with respect to the angle between planes of azole and pyrrole rings (constrained). The red dashed line is an extrapolation of the constrained lineshape to contain the freely relaxed optimized geometry and the constrained coplanar geometry at 0°.

The various components are reported in Table S2 for the four dimers considered in this work. A number of points can be deduced:

1) The reduction in the interaction energy upon adsorption is split equally between the deformation and electronic CT terms, which each comprise about 20% of the gas phase interaction energy.

2) For the CGP dimers, we further break down the deformation energy (1.8 kcal/mol in **CGP-Te**; 1.3 kcal/mol in **CGP-Se**). From Fig. S11 (left panel) and the computed Ch-N distances (Table S1) we estimate that the energy penalty due to lengthening the Ch-N bonds after matching to the substrate is about ~0.5 kcal/mol in both molecules. From Fig. S11 (right panel) we estimate the energy penalty associated with rotating the azole with respect to the pyrrole ring, from 25.5° in the gas phase to about ~12° when adsorbed, at about 0.2 kcal/mol (per fragment). Thus, the remaining penalty of ~0.9 kcal/mol in **CGP-Te** (~0.4 kcal/mol in **CGP-Se**) can be ascribed to the electrostatic and orbital misalignment (out-of-plane twisting) of the Te/Se and N atoms in the chalcogen bond induced by adsorption on the surface.

3) The vdW component in the interaction energy $\Delta E^{int,disp}_{dimer,surf}$ is fairly constant for the three dimers with Ch-N bonds. It forms a large component of the total interaction energy in the CGP-Ch dimers. It is interesting to note that despite an attractive vdW interaction present between **Benzo-Te** monomers, the

remaining non-dispersion interaction $\Delta E_{dimer,surf}^{int,non-disp}$, in the absence of chalcogen bonding, is strongly repulsive and overall destabilizes the dimer. For the smaller diazole-Te dimer, the non-dispersion contribution dominates the interaction energy.

4) Further analysis of the chalcogen bond (e.g. electrostatic, polarization, Pauli repulsion contributions) may be accessible through an energy decomposition analysis, which is however not widely available for periodic systems.

18. Molecular projected density of states (PDOS)

The projected density of states of the gas phase **CGP-Te** dimer and of one of its fragments (same geometry) is plotted in Fig. S12. A weak splitting of Te p_z orbitals is observed upon dimer formation, while a much larger splitting is observed for the in-plane Te and N molecular orbitals. These calculations quantify the overlap between Te and N orbitals in forming the chalcogen bond. The HOMO remains localized on the pyrene.

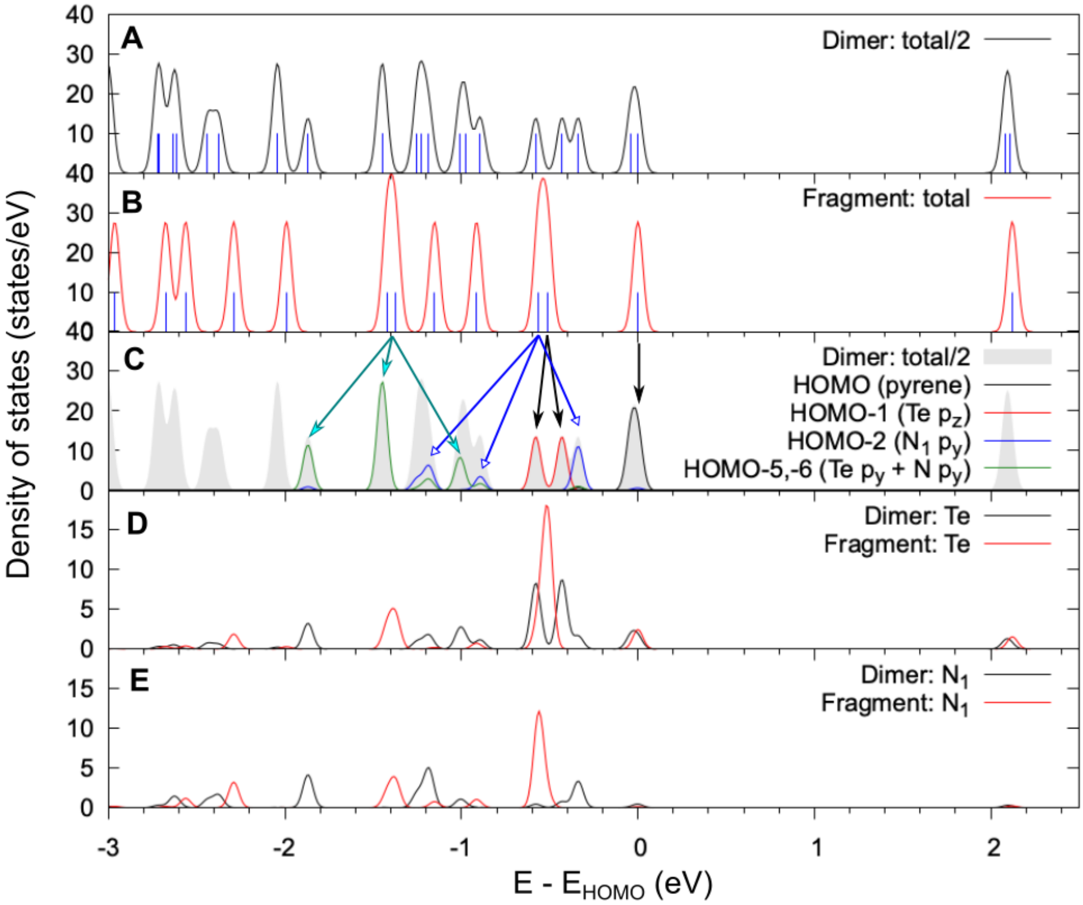

Figure S13. Projected density of states (PDOS) of CGP-Te dimer in gas phase. (A) Total PDOS of the dimer. Energy levels are indicated by vertical bars. (B) As (A), for one of the dimer fragments. (C) Molecular PDOS[6] of selected fragment states, i.e. their projection on the PDOS of the dimer. (D) PDOS for Te atom in dimer and fragment. (E) PDOS for N atom (N_1) involved in chalcogen bond, in dimer and fragment. Energies are shifted to align the HOMO in both systems.

References

1. Biot, N.; Bonifazi, D., Programming Recognition Arrays through Double Chalcogen-Bonding Interactions. *Chem. Eur. J.* **2018,** *24* (21), 5439-5443.
2. Hoffmann, M. J.; Medford, A. J.; Bligaard, T., Framework for scalable adsorbate–adsorbate interaction models. *J. Phys. Chem. C* **2016,** *120* (24), 13087-13094.
3. Michalczyk, M.; Malik, M.; Zierkiewicz, W.; Scheiner, S., Experimental and theoretical studies of dimers stabilized by two chalcogen bonds in the presence of a N··· N pnicogen bond. *J. Phys. Chem. A* **2021,** *125* (2), 657-668.
4. Scheiner, S., Principles guiding the square bonding motif containing a pair of chalcogen bonds between chalcogenadiazoles. *J. Phys. Chem. A* **2022,** *126* (7), 1194-1203.
5. Wang, H.; Li, B.; Wang, X.; Yin, F.; Wei, Q.; Wang, X.; Ni, Y.; Wang, H., First-principles study of square chalcogen bond interactions and its adsorption behavior on silver surface. *Phys. Chem. Chem. Phys.* **2023,** *25* (15), 10836-10844.
6. Ravikumar, A.; Baby, A.; Lin, H.; Brivio, G. P.; Fratesi, G., Femtomagnetism in graphene induced by core level excitation of organic adsorbates. *Sci. Rep.* **2016,** *6* (1), 24603.
